# SCAP SigFox: A Scalable Communication Protocol for Low-Power Wide-Area IoT Networks

**DOI:** 10.3390/s23073732

**Published:** 2023-04-04

**Authors:** Halah Alqurashi, Fatma Bouabdallah, Enas Khairullah

**Affiliations:** Faculty of Computing and Information Technology, King Abdelaziz University, Jeddah P.O. Box 80200, Saudi Arabia; hsalehalqurashi@stu.kau.edu.sa (H.A.); ekhairallah@kau.edu.sa (E.K.)

**Keywords:** low-power wide-area network (LPWAN), Internet of Things (IoT), SigFox technology, scalability

## Abstract

The Internet of Things (IoT) is a new future technology that is aimed at connecting billions of physical-world objects to the IT infrastructure via a wireless medium. Many radio access technologies exist, but few address the requirements of IoT applications such as low cost, low energy consumption, and long range. Low-Power wide-area network (LPWAN) technologies, especially SigFox, have a low data rate that makes them suitable for IoT applications, especially since the lower the data rate, the longer the usable distance for the radio link. SigFox technology achieves as a main objective network reliability by striving for the successful delivery of data messages through redundancy. Doing so results in one of the SigFox weaknesses, namely the high collision rate, which questions SigFox scalability. In this work, we aimed at avoiding collisions by changing SigFox’s Aloha-based medium access protocol to TDMA and by using only orthogonal channels while removing redundancy. Consequently, every node sends a single copy of the data message on a given orthogonal channel in a specific time slot. To achieve this, we implemented a slot- and channel-allocation protocol (SCAP) on top of SigFox. In other words, our goal was to improve SigFox’s scalability by implementing two mechanisms: time slot allocation and channel allocation. Performance analysis was conducted on large networks with sizes ranging from 1000 to 10,000 nodes to evaluate both technologies: the original SigFox and SCAP SigFox. The simulation results showed that SCAP SigFox highly reduced the probability of collision and energy consumption when compared to the original SigFox. Additionally, SCAP SigFox had a greater throughput and packet delivery ratio (PDR).

## 1. Introduction

The internet is continuously growing, and one of its most recent and promising advances is the Internet of Things (IoT). The IoT consists of interconnected smart devices that open new possibilities for accumulating, analyzing, and disseminating data, allowing the creation of new knowledge, and facilitating more informed decision making [1]. IoT applications are used in a range of industries, including energy, transportation, trade, agriculture, education, and healthcare [2]. In these domains, there are numerous IoT use cases, including home automation, health monitoring, and traffic management [3]. However, new IoT applications in new sectors are constantly appearing, indicating that the IoT is rapidly growing. According to [4,5], the number of IoT devices was anticipated to reach 10 billion in 2018, and this number is expected to rise to 64 billion by 2025 and 500 billion by 2030.

The technology of low-power wide-area networks (LPWANs) represents a revolutionary communication paradigm for connecting low-power end devices to the Internet of Things (IoT) [6]. LPWAN technologies have various unique characteristics such as wide-area connectivity for low power consumption, low cost, and low-data-rate devices, as well as basic network topologies such as star-of-stars [7]. SigFox and LoRaWAN are the two unlicensed-band LPWAN technologies that are now governing the market, each employing numerous techniques to guarantee long range, low power consumption, and high scalability [8]. Both technologies operate in the sub-GHz industrial, scientific, and medical (ISM) band (868 MHz in Europe and 915 MHz in the US) and employ Aloha-based media access control (MAC) with random frequency channel selection [9]. Additionally, they do not require an explicit association between an IoT device and a base station [10]. On the other hand, there are significant distinctions between the two technologies, including different bandwidths, a variety of possible modulation and coding schemes (MCS), and, finally, different constraints and limitations [11].

LoRaWAN was developed and marketed by the Semtech Corporation [12] and is a physical layer technology that utilizes a patented spread spectrum approach to modulate signals in the sub-GHZ ISM band [13]. A unique chirp spread spectrum (CSS) technology distributes a narrow band input signal over a broader channel bandwidth to enable bidirectional communication. The resultant signal has noise-like characteristics, making it more difficult to jam or detect. Resilience to noise and interference is made possible by the processing gain [14].

SigFox is a France-based LPWAN technology that serves as both a network operator and a technology standard [15]. The patented base stations with cognitive software-defined radios are installed by SigFox network operators (SNOs) who use an IP-based network to link them to the backend servers. IoT devices communicate with these base stations through binary phase shift keying (BPSK) modulation over an ultra-narrow (100 Hz) sub-GHZ ISM band carrier (UNB). UNB allows SigFox to efficiently use bandwidth and experience very low noise levels, which leads to a high receiver sensitivity, low power consumption [16], and low-cost antenna design [17].

Based on the performance analysis derived in [18] where a comparison between LoRaWAN and SigFox was conducted from an MAC perspective, the outcomes of the MATLAB simulation demonstrated that SigFox has a broad coverage and can accommodate more devices, which makes SigFox’s scalability greater than that of LoRaWAN. Indeed, SigFox is used in more than 70 countries [19] and has a range of up to 40 km [20]. However, in some scenarios where the number of nodes is extremely high, high collision rates may be experienced in SigFox. This is why SigFox’s scalability must be investigated in order to further increase the number of supported nodes.

The main shortcoming of SigFox is the use of an Aloha-based medium access protocol alongside a redundancy mechanism. Indeed, despite its simplicity, the performance of Aloha-based medium access deteriorates with the increase in the number of connected devices, since collisions will inevitably increase. Moreover, the fact that SigFox needs to transmit three copies of the same packet to guarantee reliability can cause spectrum congestion and increase the collision rate dramatically as the number of IoT devices increases, as proven in [21]. Moreover, SigFox uses 1920 partially overlapping channels, which makes it even more susceptible to collision [13]. Indeed, SigFox uses a sub-GHz band with a width of 192 kHz in the European band, resulting in 1920 partially overlapping channels with a width of 100 Hz each, where only 360 of them are orthogonal. For these reasons, by adopting TDMA access along with carefully assigning only the subset of the orthogonal channels as well as avoiding redundancy to the highest possible extent will inevitably further enhance SigFox’s reliability. 

The key contributions of this paper are listed as follows:

1. Changing the medium access protocol of the original SigFox from Aloha to time division multiple access, TDMA.
2. Using only the orthogonal channels of SigFox to transmit data to the gateway.
3. Avoiding redundancy by transmitting a unique copy of the message.
4. Designing an autonomous conflict-free slot-assignment procedure that defines the TDMA frame size and allows every sensor to autonomously select its slot identifier.
5. Conceiving an autonomous channel allocation procedure that allows every sensor to autonomously determine its transmission channel such that nodes using the same slot identifier are on different channels. Thus, several simultaneous communications may take place without collisions.

It is worth pointing out that the main reason behind designing the slot assignment and channel allocation procedures is to mitigate the collision to improve the network throughput and hence further enhance the network’s scalability. Moreover, the autonomous features of both procedures first allow further improvements to the energy efficiency by avoiding extensive communication with the network server through the gateway. Most importantly, the duty cycle constraint on the ISM band dramatically compromises the downlink communication between the gateway and the sensor nodes. Consequently, any centralized decision on the gateway side is barely disseminated to end devices as it may take a very long time. Consequently, opting for autonomous procedures will avoid duty-cycled communication with the gateway, and hence faster network convergence to the optimal configuration can be achieved. Therefore, collisions and energy consumption can be further improved. Note that in duty-cycled networks, distributed autonomous solutions are preferred over centralized ones due to their energy efficiency and faster convergence, especially if they are perfectly designed.

The rest of this paper is organized as follows: Section 2 presents the background of SigFox. Section 3 reviews previous work related to SigFox. Section 4 states our research problem. Section 5 describes the proposed slot- and channel-allocation protocol (SCAP). Section 6 evaluates the performance of SCAP compared to the original SigFox. Finally, Section 7 draws our conclusions.

## 2. Background

In IoT networks, numerous communication technologies can be utilized to offer a wireless connection, such as SigFox. SigFox technology is an end-to-end LPWAN technology that is employed for several IoT applications. SigFox technology is used to offer network connectivity services for IoT networks as it is classified as a low-throughput network (LTN) protocol [22]. 

### 2.1. SigFox Architecture

**Figure 1:**
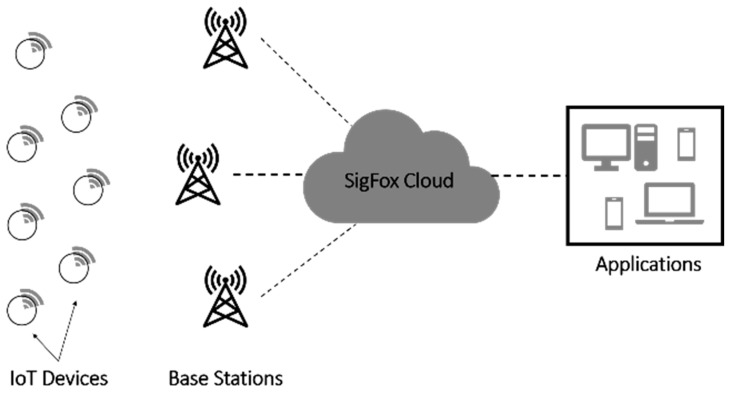
SigFox components.

SigFox has three main components: IoT devices, SigFox base stations (gateways), and the SigFox cloud, [15] as shown in Figure 1.

IoT devices transmit messages to base stations that then forward these messages toward the SigFox cloud through an IP-based network. In the SigFox cloud, the messages are processed and then sent to be screened by customer servers. Furthermore, the SigFox cloud provides several services such as map predictions and user account management [13]. SigFox uses a star topology and Aloha as its medium access protocol, which is a random-access MAC protocol, along with random channel selection. Moreover, according to SigFox, no explicit association between the IoT devices and the base station is required as opposed to cellular networks. Therefore, no synchronization mechanism is needed, which can lower its energy consumption and cost [23].

### 2.2. SigFox Technical Features and Goals

SigFox is designed so that the IoT device’s battery can live up to 6.5 years with a maximum transmission power of 14 dBm [21]. SigFox operates in an unlicensed industrial, scientific, and medical (ISM) sub-GHz band (below 1 GHz) with a 1% duty cycle. Moreover, SigFox shrinks the signal bandwidth to improve the receiver’s sensitivity and reduces the in-band noise. IoT SigFox devices use differential binary phase shift keying (D-BPSK) modulation to send data to the base station in an ultra-narrow band signal with a signal band of approximately (100 Hz) [17]. The maximum data rate is 100 bps.

### 2.3. Communication and Dataflow

The SigFox base station permanently listens to the spectrum to receive messages and forward them to the SigFox cloud. SigFox is a bi-directional technology where two types of communication with very small messages are provided: uplink communication with 140 messages/day and a maximum payload of 12 bytes and downlink communication with 4 messages/day and a maximum payload of 8 bytes. This increases the network capacity and provides a high transmission efficiency [10].

For reliability purposes, SigFox uses a redundancy mechanism to increase the packet’s chance of successfully reaching the base station. Indeed, SigFox employs the following:

(1) Time diversity, where the IoT device transmits the same data message on three different random time offsets.
(2) Frequency diversity, where the IoT device transmits the same data message on three different random frequencies (channels).
(3) Spatial diversity, where the IoT-transmitted packets should be received by three different base stations as there is no explicit association to any given base station [24].

Despite SigFox’s currently achieved performance, there is still room for improvement since SigFox suffers from high collision due to its Aloha-based medium access protocol along with its redundancy mechanism, as proven in [21]. Indeed, for simplicity reasons, SigFox uses an Aloha-based communication protocol with a redundancy mechanism to ensure successful delivery to the base station. However, doing so increases the likelihood of collision, since IoT devices transmit the same packet three times without listening to the channel. Consequently, SigFox is susceptible to high collision rates, since various IoT devices may transmit data simultaneously. As expected, collisions decrease the network’s capacity due to the loss of data and, hence, the network’s scalability is constrained. 

Furthermore, collisions necessitate retransmissions, which reduce the network’s throughput as the data packet end-to-end delay increases. The number of devices, the size of the transmitted messages, and the communication speed are all factors influencing the likelihood of a collision. The ever-increasing number of devices in IoT networks makes them particularly susceptible to collisions, making collisions a problem that must be addressed to further improve network scalability [24]. 

There have been studies conducted to reduce the number of collisions in SigFox networks [5,25,26]. However, it should be mentioned that most of the proposed solutions to enhancing SigFox are centralized, which not only creates an operating cost in the base station but also results in slow and hard network convergence to optimality, as communication in the sub-GHz ISM band are duty-cycled. For these reasons, our main goal was to propose a distributed solution where each node autonomously selects its optimal transmission parameters in terms of slot and channel identifiers without any extra packet exchange between the IoT device and the base station. This is different to previously proposed research works where the network server is in charge of not only computing the optimal transmission settings for every node but also disseminating it in a harshly duty-cycled network.

## 3. SigFox-Related Work

**Figure 2:**
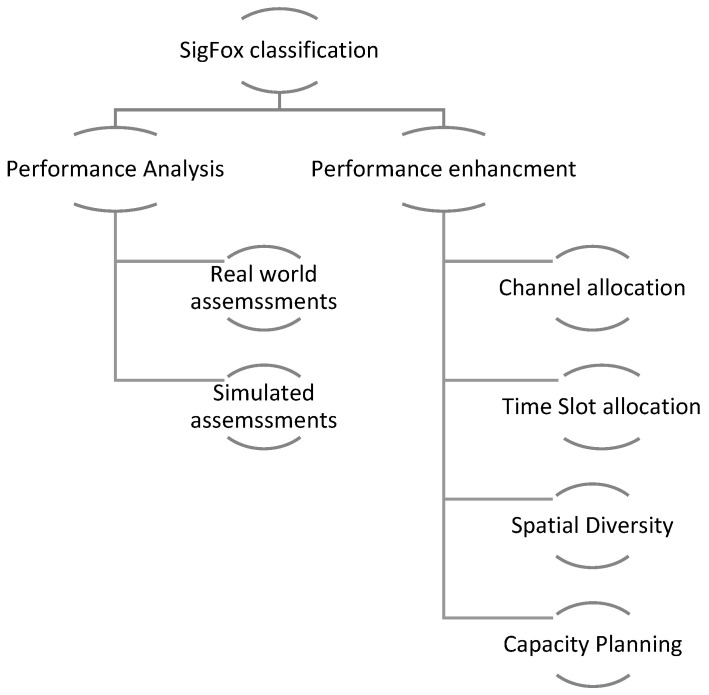
SigFox-related work classification.

In this paper, work related to SigFox technology is classified into two categories, namely SigFox performance analysis and SigFox performance enhancement. In the first category, we present recent works that focus on analyzing SigFox network performance as an LPWA technology, while in the second category, we describe the new solutions that aim at improving SigFox’s performance. Moreover, the two main categories are divided into sub-categories for efficient classification. Figure 2 shows the full classification of the work related to SigFox. 

### 3.1. SigFox Performance Analysis

#### 3.1.1. Real-World Assessments

In [14], Mikhaylov et al. presented an assessment of the real-world communication performance and radio channel properties of a large-scale SigFox network in 311 different test locations. The assessment was conducted over three months in the city of Brno, Czech Republic. Furthermore, the RSSI-based localization performance from multiple SigFox BS was demonstrated, and a propagation model was determined. The communication performance was assessed in a 12 km north-to-south and 24 km west-to-east area, which was served by multiple SBS-T3 base stations. As the IoT device equipment, an Adeunis RF SigFox field test device was used. Each IoT device was located almost one meter above the ground far from buildings and transmitted messages in multi-frame mode through the uplink procedure. The beginning and ending timestamps of each experiment in addition to its precise site based on the global navigation satellite system (GNSS) receiver were validated. As a result, they showed that 95.5% of the messages were received successfully. Moreover, the cumulative packet delivery ratio (PDR) reached 94.79%, and an average of 3.56 base stations received the same packet. As for the RSSI-based localization performance, the measured RSSI data were used to determine the test point’s location. As mentioned, SigFox offers native support for geolocation based on RSSI and machine-learning, but feature specifics are not available. Therefore, two conventional methods were explored: 

Method 1: assigning the base station coordinates with the highest RSSI as an estimate of the test point site. The average localization error verified over all the packets was 3.54 km, whereas the maximum and minimum errors were 25.5 and 0.13 km, respectively. However, the error was lower than 5.28 km for 80% of the packets.

Method 2: conventional triangulation, where the packets that have been received by at least three base stations were located; if more than three base stations received the packet then the three base stations with the highest RSSI were used. The average error was 6.31 km, whereas the maximum and minimum errors were 14.49 and 1.40 km, respectively. However, the error was lower than 8.52 km for 80% of the packets.

#### 3.1.2. Simulated Assessments

In [21], Lavric et al. evaluated and analyzed SigFox’s performance level. This work emulated a local SigFox base station with the use of a SigFox SDR dongle. This tool was developed to promote the developer to realize the initial SigFox conformity assessments. The module could function in a spectrum analyzer configuration, whose functionality was developed using the Ubuntu Linux operating system. To complete the setup, a software application was designed, implemented, and tested. Its role was to run on the ON SigFox-GEVB evaluation board module. As a result, it was proven that SigFox technology is a convenient candidate for implementing IoT concepts. However, it suffers from high collision, especially in extremely large IoT networks.

In [27], Osman et al. evaluated SigFox’s performance by using a Monte Carlo simulation method. This simulation evaluated the impact of the number of IoT devices on the performance of the SigFox network by measuring each of the following metrics: spectrum efficiency, collision, and packet error rate (PER). The Monte Carlo simulation method creates suitable random numbers and observes the numbers that comply with some property to solve different problems. The goal is to observe the impact of the number of IoT devices on the performance metrics. As a result of these experiments, they showed that the packet collisions increased exponentially when the traffic rate increased, and the packet error rate (PER) increased when the number of IoT devices increased.

In [13], Lavric et al. presented a SigFox communication model with a performance evaluation using an implemented SigFox traffic generator with software-defined radio (SDR). Three scenarios were evaluated using all 1920 channels, where every message was transmitted on three different communication channels. It was shown that the performance level was highly impacted by the total number of SigFox devices. Furthermore, two other scenarios were evaluated, with the total number of IoT devices varying between 200–3000 and 1000–10,000, respectively. One scenario considered the packet error rate (PER) parameter when transmitting the packet using three random frequencies at different time intervals, and the other scenario involved transmitting the packet only once using one single frequency channel. As a result, when the packets were transmitted using one single channel, the packet error rate (PER) was approximately 14% lower than when the packets were transmitted on three different frequencies, which showed the inefficiency of the channel redundancy when the number of IoT devices was extremely high.

In [9], Lalle et al. presented a scalability analysis between three LPWAN technologies (SigFox, LoRaWAN, and NB-IoT) using a discrete event network simulator (NS3). Three cases were studied with a configurable number of IoT devices (100, 500, 1000, 2000, 5000, 7000, 10,000, 15,000, or 20,000) and a simulation time equal to 6000 s. Case one involved one base station, case two involved two base stations, and case three involved four base stations. As a result, it was concluded that the number of base stations had a great impact on the network’s performance, and there was a positive correlation between the number of IoT devices and the packet error rate (PER).

In [28], Osman et al. presented an evaluation of the performance of SigFox technology by using a MATLAB simulation model. In this simulation, the performance of SigFox was evaluated in terms of the collision, packet error rate (PER), and spectrum efficiency using different values for the channel bandwidth. Furthermore, an investigation on how the number of IoT devices impacts SigFox network performance was conducted. As a result, it was concluded that increasing the number of IoT devices resulted in increasing the packet collision and PER.

### 3.2. SigFox Performance Enhancement

#### 3.2.1. Channel Allocation

In [5], G.C. et al. proposed a radio resource management (RRM) framework based on a software-defined network (SDN), which allows efficient radio resource allocation by using an SDN-centralized controller. The RRM framework consisted of three components: IoT devices, IoT base stations, and an SDN controller. The SDN controller itself included three important components: a spectrum sensing module, a channel status database, and a channel allocation module. The SDN controller was directly connected to a set of base stations to monitor and collect network information through the uplink control channels. These modules analyzed and calculated the optimal channel allocation to avoid transmission collisions between the IoT devices. As an advantage, the resource contention was reduced, and the RRM framework provided coordinated communications. As for its limitations, there was a higher level of computational complexity at the central node, which was the SDN, and there was a high probability of simultaneous access to the same channel, which will result in collisions.

#### 3.2.2. Time Slot Allocation

In [25], Pullmann et al. proposed a slot-based communication planning protocol (SCPP), where gateways periodically planned the communication by assigning IoT devices a specific time slot from the transmission schedule to avoid collisions. The slot-based communication planning protocol (SCPP) is a control protocol for communication scheduling that is used as a collision-prevention technique. Two types of communication exist: planned communication, which involves periodic transmission during reserved time slots for a specific IoT device, and unplanned communication, which involves unexpected communication from any IoT device. Furthermore, five key functions were introduced into the SCPP protocol: time slot reservation, time slot revocation, data transmission, time synchronization, and parameter propagation. It is true that the collision probability and number of retransmissions were reduced, but some time slots were still shared between the IoT devices, so collisions may occur. Other limitations of this work include its implementation complexity and higher control overhead, especially at the gateway side. Finally, we noticed that the channel or frequency diversity was neglected.

In [26], Tsoukaneri et al. proposed an on-demand scheme as an alternative scheme to the single-cell point-to-multipoint (SC-PTM) framework, which is an approach to expanding multimedia broadcast multicast services (MBMSs) and to providing group communications inside a single cell. This scheme is more efficient from the perspectives of bandwidth usage and device energy consumption. In this scheme, when a multicast message is to be sent, the evolved node alerts the IoT devices of the upcoming multicast message so that the IoT devices do not require periodic wake-ups to check for possible multicast messages. As a result, the scheme reduces the signaling overhead of the node and the energy consumption. However, the authors did not discuss how the IoT devices could be synchronized to wake up at the same time and receive the multicast message with a single transmission.

#### 3.2.3. Spatial Diversity

In [29], Mo et al. presented interference cancellation and signal-combining technologies across multiple base stations to achieve an optimal outcome by taking advantage of SigFox’s spatial diversity. First, the application of signal-combining technologies involved selection combining (SC), which aims at choosing the optimal quality signal of all base stations, and equal fain combining (EGC) or max ratio combining (MRC), which both aim at maximizing the output’s signal interference ratio (SINR). Second, they applied signal interference cancellation technologies either locally and/or globally to reconstruct and retrieve the contributions of decoded packets with an iterative success interference cancellation (SIC) procedure. As expected, the global technologies taking advantage of multiple base stations had better performances than the local single base station ones. Moreover, max ratio combining (MRC) and/or equal gain combining (EGC) had a better performance than the selection combining (SC) procedure. Finally, max ratio combining (MRC) outperformed the equal gain combining (EGC) technology. Unfortunately, these interference cancellation and signal-combining technologies increase the implementation complexity, which results in a higher cost and power consumption.

#### 3.2.4. Capacity Planning

In [30], Febriyandi et al. presented a SigFox radio frequency (RF)-network-planning analysis for IoT services in a metropolitan area with a population of 10.37 million and an area of 662.3 ${km}^{3}$. This analysis was conducted to form an enhanced SigFox network concerning capacity planning. The Okumura–Hate radio propagation model was used as the path loss modeling, and the ATOLL software was used as a tool to process the data. The planning analysis followed the following sequence: defining SigFox radio parameters, which must reflect the technical specifications of the Ministry of Communication and Information (MCIT), determining which type of base station and antenna is to be used, budget linking and coverage planning using the Okumura–Hate propagation model, and, finally, the ATOLL software was used to run the SigFox network planning program with the specific parameters obtained previously. As a result, the optimal number of base stations required to cover a certain area alongside their appropriate locations is obtained so as to improve the scalability of SigFox. 

Based on recent work on SigFox, it has been proven that SigFox suffers from a high collision rate, especially when the number of IoT devices increases, which limits SigFox’s scalability, as shown in [27]. It is true that some related studies [5,25,26] have proposed new solutions, including radio resource management or time slot allocation, to improve SigFox’s performance. However, these solutions add extra computational complexity as well as provide a bottleneck at the base station due to the centralized procedures. Indeed, using centralized procedures is questionable for SigFox networks, as the downlink communication is also subject to the 1% duty cycle constraint. Consequently, keeping the optimal configuration of nodes to the network server will take a huge amount of time, especially for large-scale IoT networks where the number of nodes is expected to be in the billions. Hence, the network will operate under sub-optimal settings even if the optimal configuration for every node has been already computed by the network server, as the network convergence to optimality is so slow. 

For these reasons, and as the main contribution of our work, we propose a distributed solution where the nodes autonomously compute their optimal communication settings by knowing only their coordinates as well as those of the base station. Accordingly, two main autonomous mechanisms are used to enhance the network capacity and performance of SigFox technology: a channel-assignment mechanism and a time-slot-assignment mechanism. In the latter, the Aloha-based access of the original SigFox is changed to a TDMA-based one in order to reduce the channel contention. Hence, every node autonomously determines its slot identifier without any notification from the base station while guaranteeing conflict-free timeslot selection. Similarly, for the channel-assignment mechanism, each IoT device autonomously calculates its channel identifier without any communication with the base station to overcome the duty-cycled communication in the ISM band while also ensuring conflict-free channel selection. 

Furthermore, in order to further reduce the number of collisions as well as the energy consumption, we opted for using only the 360 orthogonal channels to transmit a unique single copy of every message as opposed to the redundancy scheme used in the original SigFox.

## 4. Problem Statement

SigFox technology is designed to achieve a long range and a low power consumption at the expense of high latency [16]. The main concept behind SigFox technology is its frequency and time diversity, which states that each IoT device transmits a message three times on different random time offsets and different frequencies, ensuring that if one transmission is lost or disrupted due to interference, one of the other two will be delivered successfully [15]. In other words, SigFox relies on message redundancy to ensure high reliability, which increases the network traffic. This redundancy mechanism has been shown to dramatically affect the network performance in terms of collision probability and throughput, especially when the number of IoT devices increases [13]. Furthermore, due to frequency and time diversity mechanisms, energy consumption is higher in SigFox than in other LPWAN technologies [31]. The main cause of this energy surge is the message redundancy, which is reduced in our SCAP SigFox solution [32].

From another aspect, the SigFox spectrum bandwidth in Europe is 192 kHz, and the channel width is 100 Hz, which results in 1920 available channels where only 360 channels among them are orthogonal [22]. Indeed, in SigFox, due to the Aloha-based access, the highest number of IoT devices that can communicate simultaneously while guaranteeing a high level of performance is approximately 100 when only the 360 orthogonal channels are used [13] due to the Aloha-based access. For this reason, we aimed at increasing the number of successfully simultaneously communicating IoT devices to 360 by first adopting a TDMA channel access mode instead of the Aloha-based one and, second, by carefully assigning the orthogonal channels while removing the packet transmission redundancy. By doing so, we aimed at improving the network’s energy efficiency as well as its reliability and scalability. 

Another apparent issue states that the number of packet collisions and retransmissions in SigFox increases when the number of IoT devices increases mainly due to the Aloha-based access on overlapping channels along with the redundancy mechanism [24]. The main challenge of this paper was to overcome these limitations and create a valuable solution to improve the handling of the increase in the number of IoT devices without discarding the main concepts of LPWAN technologies, which include: low power consumption, low cost, and long range. The proposed slot- and channel-allocation protocol (SCAP) is an autonomous collision-prevention protocol that can ensure the reliability of the network while reducing the collision rate in order for SigFox to be a scalable and reliable solution. We should mention that SCAP SigFox cannot completely mitigate collision and retransmission in extremely large networks if simplicity is to be maintained; however, it would highly reduce them.

## 5. Protocol Description

The slot- and channel-allocation protocol (SCAP) is ideally suited to monitoring natural applications where the communication is predictable. In such scenarios, factors such as the periodicity, transmission duration, and data size are predetermined. 

The slot- and channel-allocation protocol (SCAP) is intended for long-range IoT networks in which IoT devices communicate regularly and where the communication is centralized via network access points (base stations). The slot- and channel-allocation protocol (SCAP) mainly targets uplink communication, which refers to the messages sent from the IoT devices to the base stations.

The slot- and channel-allocation protocol (SCAP) is an autonomous collision-prevention protocol that aims at increasing the number of simultaneous and successfully communicating IoT devices to 360 devices without requiring any energy- or time-consuming procedure from the gateway. In other words, the goal is to mitigate the number of collisions to the highest possible extent in extremely large networks to improve the network’s reliability and scalability. Indeed, in the original SigFox, the highest number of IoT devices that can communicate simultaneously while guaranteeing a high level of performance is approximately 100 when only the 360 orthogonal channels are used.

The slot- and channel-allocation protocol (SCAP) improves SigFox’s scalability, throughput, packet delivery ratio (PDR), and energy consumption by adopting two mechanisms, namely, the orthogonal channel self-assignment procedure and TDMA-based channel access with the careful selection of the frame size and the autonomous assignment of slots.

### 5.1. Channel Allocation for IoT Devices 

Our channel allocation mechanism is a radio resource management scheme that uses only the orthogonal channels of the original SigFox channels. As aforementioned, in SigFox, the number of available channels is restricted to 1920 channels because the bandwidth in Europe is 192 kHz and the channel width is equal to 100 Hz. Due to the distinct frequency shift of each SigFox module, this channel number is theoretically calculated. However, adjacent channels should be avoided to achieve a higher performance and a low packet error rate (PER), as they are partially overlapping. Consequently, only 360 channels are completely orthogonal. 

**Figure 3:**
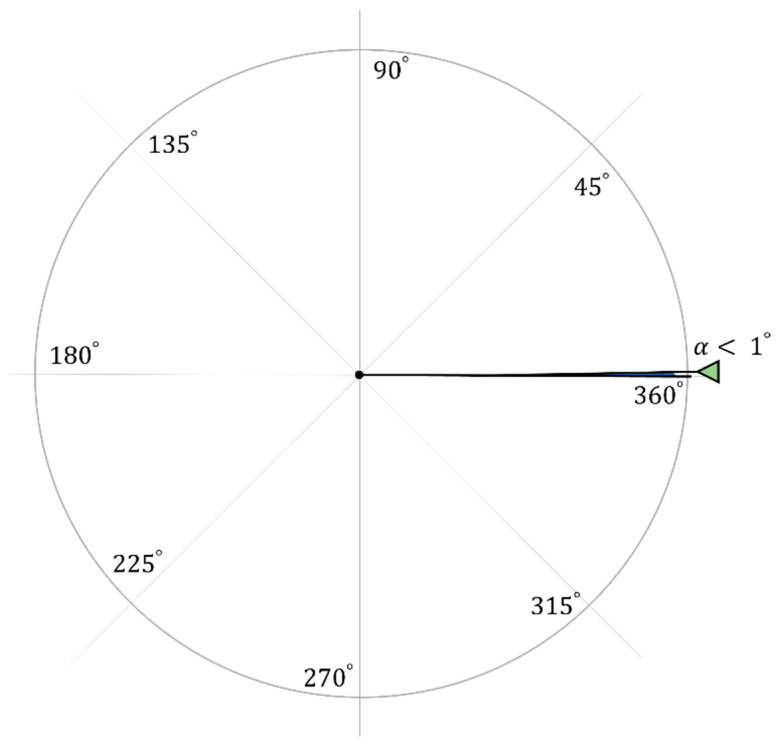
The sector angle of SCAP.

In our study, we considered a circular network field where the base station is located at the center. As shown in Figure 3, our field was divided into sectors, each with an angle α, where the nodes in the same sector are assigned the same channel. Indeed, if M channels are to be used, then α can be simply written as follows:

(1) α=2π/M,

As we targeted autonomous channel allocation, each node must be able to identify the sector to which it belongs without any communication with the base station. To achieve this, we supposed that each node knows its coordinates as well as those of the base station. Three steps are then followed by the IoT device to determine its sector identifier and hence that of its channel.

**Figure 4:**
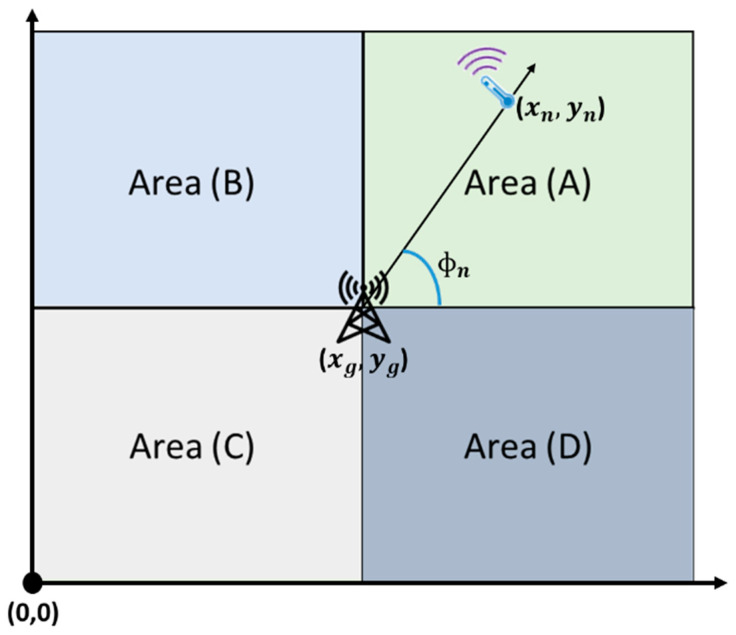
SCAP SigFox area categorization.

Step 1: using its coordinates, each IoT device pinpoints the area it exists in relative to the base station. As shown in Figure 4, area (A) represents the top-right corner, area (B) represents the top-left corner, area (C) represents the bottom-left corner, and area (D) represents the bottom-right corner. 

Please note that in our study, the origin of our field was the bottom left corner of area (C), as shown in Figure 3. To find its area, the IoT device, n, using its coordinates ($x_{n},y_{n}$) as well as the base station coordinates ($x_{g},y_{g}$), simply checks the following: (i) if $x_{n}>x_{g}$ and $y_{n}>y_{g}$, then it belongs to area (A), (ii) if $x_{n}<x_{g}$ and $y_{n}>y_{g}$, then it belongs to area (B), (iii) if $x_{n}<x_{g}$ and $y_{n}<y_{g}$, then it belongs to area (C), and finally (iv) if $x_{n}>x_{g}$ and $y_{n}<y_{g}$, then it belongs to area (D).

Step 2: each IoT device, n, calculates (${ɸ}_{n}$), which is the exact angle between the IoT device and the base station using one of the following equations based on the previously defined areas:

$$\begin{array}{cc} \mathrm{Area}\;(A):\; & {ɸ}_{n}=\mathrm{atan}\left(\frac{y_{n}-y_{g}}{x_{n}-x_{g}}\right)\\ \mathrm{Area}\;(B):\; & {ɸ}_{n}=\pi -\mathrm{atan}|\left(\frac{y_{n}-y_{g}}{x_{n}-x_{g}}\right)|\\ \mathrm{Area}\;(C):\; & {ɸ}_{n}=\pi +\mathrm{atan}|\left(\frac{y_{n}-y_{g}}{x_{n}-x_{g}}\right)|\\ \mathrm{Area}\;(D):\; & {ɸ}_{n}=2\pi -\mathrm{atan}|\left(\frac{y_{n}-y_{g}}{x_{n}-x_{g}}\right)| \end{array}$$

where ${ɸ}_{n}$ is the relative angle of the IoT device to the gateway, ($x_{n},y_{n}$) is the IoT device’s coordinates, and ($x_{g},y_{g}$) is the base station’s coordinates.

Step 3: since the slot- and channel-allocation protocol (SCAP) uses only a subset, M, of the total number of available channels, namely the orthogonal ones, each IoT device computes its channel identifier using Equation (2):

(2) $$n_{channel}=\frac{{ɸ}_{n}}{\alpha },$$

where $n_{channel}$ is the channel identifier of the IoT device n.

Please note that since we aimed at using all the orthogonal channels, M = 360 and, hence, α=2π/360. It is worth noting that the small value of α helps to reduce the TDMA frame size as the area of the sector of angle α is so small, as will be explained in the next Section 5.2.

### 5.2. Time Slot Allocation for IoT Devices

Since the nodes in the same sector use the same channel, collisions among them may happen if we keep the Aloha-based access mode. Thus, collisions will be further avoided using TDMA-based access. Consequently, we need to decide the TDMA frame size as well as the mechanism of assigning time slots to nodes. It is worth noting that by adopting TDMA, a single copy of the message is transmitted. In other words, there is no need to overwhelm the network with redundant messages just to ensure successful delivery, as every node has its own slot and channel to guarantee conflict-free communication.

The slot-and-channel-allocation protocol (SCAP) divides continuous time into frames where each frame consists of a fixed number of time slots that can be used to transmit messages using IoT devices. According to our proposed autonomous time-slot-allocation procedure, the frame size (m) can be derived using Equation (3). Accordingly, m is estimated to be the average number of sensors in a line of length R. Since α has a very small value, we can fairly and reasonably assume that the maximum number of nodes in every sector will not exceed m since the sector area is almost a line, as shown in Figure 3.

Accordingly, the maximum frame size is proportional to the network field size and the network density. Indeed, the maximum frame size m is expressed as follows:

(3) $$m=\frac{R}{d}$$

where R is the network radius and d is the average distance between the IoT devices. Note that to find d, i.e., the average distance between the IoT devices in the network, the network density (P) must be computed using Equation (4):

(4) $$P=\frac{N}{A}$$

where N is the total number of IoT devices and A is the network area (network surface). Indeed, A simply equals $\pi R^{2}$.

Once the network density (P) is calculated, the average distance between the IoT devices (d) can be computed using Equation (5):

(5) $$d=\sqrt{\frac{1}{P}}$$

Consequently, using Equations (3)–(5), the maximum frame size is determined. Now, each IoT device in the network determines its time slot based on the distance between its location and the base station’s location. This slot identifier cannot be greater than m and is derived using Equation (6).

(6) $$n_{slot}=\left(\frac{d_{nG}}{d}\right)+1$$

where $n_{slot}$ is the time slot identifier of the IoT device n, $d_{nG}$ is the distance between the IoT device and the gateway (base station), and d is the average distance between the IoT devices. 

It is worth noting that our slot-assignment procedure and the orthogonal channel-assignment procedure mutually collaborate to guarantee, to the highest possible extent, collision-free communication in large-scale networks. Indeed, each IoT device n autonomously predetermines its slot identifier ($n_{slot})$ as well as its channel identifier ($n_{channel}$). Thus, once the IoT device generates a message it transmits it only on the assigned slot and channel. In this way, rather than transmitting randomly, the transmission process can be controlled to improve the network’s performance in terms of scalability, reliability, and energy efficiency. The slot- and channel-allocation protocol (SCAP) ensures that even if two or more IoT devices have the same time slot, the message will be transmitted successfully due to the allocation of different channel identifiers and vice versa. 

**Figure 5:**
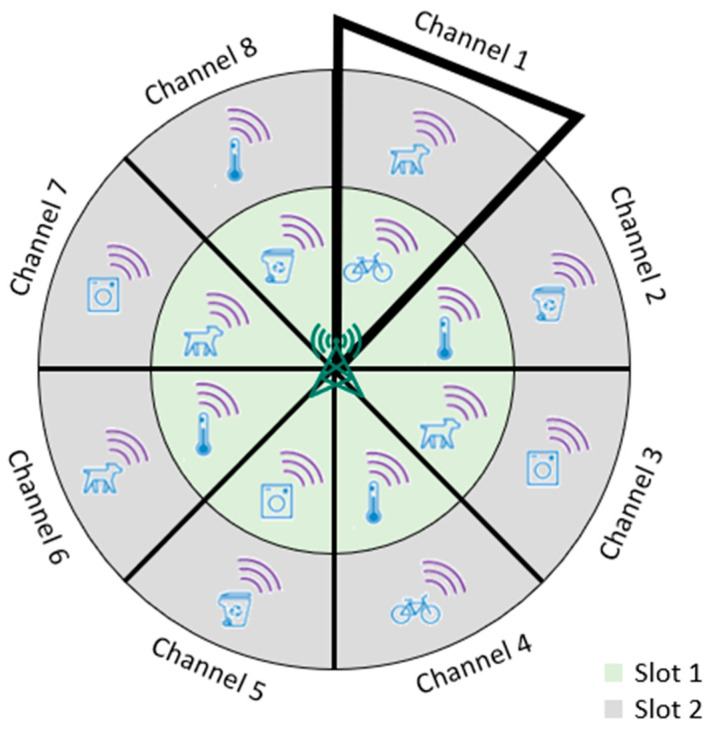
The basic principle of the slot- and channel-allocation protocol (SCAP).

Figure 5 illustrates the basic principle of the slot- and channel-allocation protocol (SCAP) mechanism. Please note that due to the large number of available orthogonal channels, in Figure 5 only eight channels and two slots are depicted as a simple illustrative example. According to Figure 5, although all the IoT devices in the inner green circle have the same time slot (1), they do not collide with each other since they are assigned different channel identifiers (Channel 1–Channel 8). In other words, each IoT device can transmit its message during the assigned time slot and using its channel identifier without collisions. This indeed reduces the number of collisions and increases the throughput and the packet delivery ratio (PDR).

**Figure 6:**
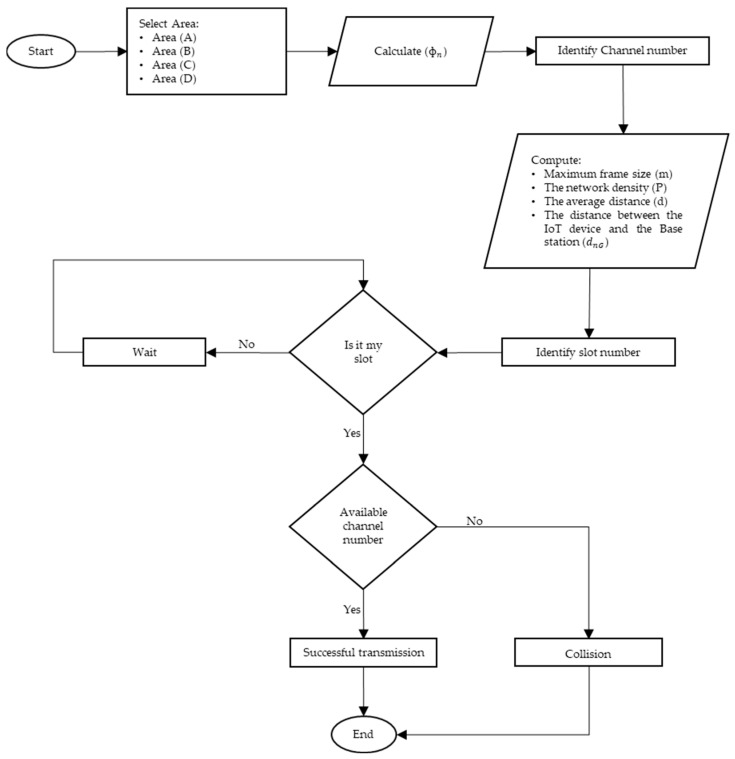
The workflow of the slot- and channel-allocation protocol (SCAP).

For a better understanding of the protocol operation, Figure 6 describes the workflow of the mechanisms of the slot- and channel-allocation protocol (SCAP). Accordingly, it shows the flow of each step, as described previously, in SCAP in order to send a packet. Moreover, Algorithm 1 shows the algorithm sequence of the slot- and channel-allocation protocol (SCAP).
**Algorithm 1** The algorithm sequence of the slot- and channel-allocation protocol (SCAP)1.**Set M**
2.Calculate α=2π/M

3.Obtain ($x_{n},y_{n}$) and ($x_{g},y_{g}$), for each IoT device
4.**if** ($x_{n}-x_{g}$) > 0 and ($y_{n}-y_{g}$) > 0 **then**
5.    Calculate ${ɸ}_{n}=\mathrm{atan}\left(\frac{y_{n}-y_{g}}{x_{n}-x_{g}}\right)$

6.**else if** ($x_{n}-x_{g}$) < 0 and ($y_{n}-y_{g}$) > 0 **then**
7.   Calculate ${ɸ}_{n}=\pi -\mathrm{atan}|\left(\frac{y_{n}-y_{g}}{x_{n}-x_{g}}\right)|$

8.**else if** ($x_{n}-x_{g}$) < 0 and ($y_{n}-y_{g}$) < 0 **then**
9.   Calculate ${ɸ}_{n}=\pi +\mathrm{atan}|\left(\frac{y_{n}-y_{g}}{x_{n}-x_{g}}\right)|$

10.**else if** ($x_{n}-x_{g}$) > 0 and ($y_{n}-y_{g}$) < 0 **then****Symbols****Descriptions**11.    Calculate ${ɸ}_{n}=2\pi -\mathrm{atan}|\left(\frac{y_{n}-y_{g}}{x_{n}-x_{g}}\right)|$
**M**The number of orthogonal channels.12.**end if**αThe sector’s angle.13.Obtain $n_{channel}$, for each IoT device**(**$x_{n},y_{n}$**)**The coordinates of the IoT device n.14.Set m, P, and d**(**$x_{g},y_{g}$**)**The coordinates of the base station.15.Obtain $d_{nG}$, for each IoT device${ɸ}_{n}$The IoT device relative angle to the base station.16.Obtain $n_{slot}$, for each IoT device$n_{channel}$The channel identifier of the IoT device n.17.**for** each IoT device N = 1, 2 …, N **do**
**m**The maximum frame size.18.    **if** IoT device has a packet to transmit **then****P**The network density.19.      Execute SCAP SigFox**d**The average distance between IoT devices.20.    **end if**$d_{nG}$The distance between IoT device n and the base station.21.**end for**$n_{slot}$The slot identifier of the IoT device n.

## 6. Performance Evaluation

In this section, the performance of SigFox and SCAP SigFox are evaluated in an IoT network simulator. In order to compare the performance of SCAP SigFox to the original SigFox, an implementation of the two models was conducted using the MATLAB platform. 

The MATLAB simulation was conducted using the available original SigFox code from [13]. Furthermore, an implementation using the parameter settings shown in Table 1 was conducted in order to compare our SCAP solution with the available original SigFox implementation.

In both models, several numbers of devices varying from 1000 to 10,000 were deployed randomly within the transmission range of the base station, resulting in a circular network field. Although in the simulation of SCAP the bandwidth was set to 192 kHz and the channel size was 100 Hz, the number of used channels was restricted to 360, which corresponded to the orthogonal channels. Moreover, the transmission time was set to 2 s, the payload of a message was 96 bits, and the transmission power was equal to 25 mW [13].

In the original SigFox, the Aloha media access technique is employed along with a random selection of channels [30]. In other words, each IoT device transmits three copies of the same message at random time intervals and on random channels. 

In contrast, in SCAP SigFox, the time division multiple access (TDMA) along with the proposed slot and channel assignment procedures were implemented to improve the network’s performance. In SCAP SigFox, the IoT device transmits only one message after predetermining its time slot and channel identifiers. The SCAP SigFox solution is based on the IoT device’s exact location as well as the base station’s location. Each IoT device autonomously computes its slot identifier and its channel identifier without any intervention or communication between the IoT device and the base station. This means that no extra messages need to be exchanged between the IoT device and the base station, which not only respects the duty cycle constraint but also saves energy and time while being rapidly optimal. 

**Figure 7:**
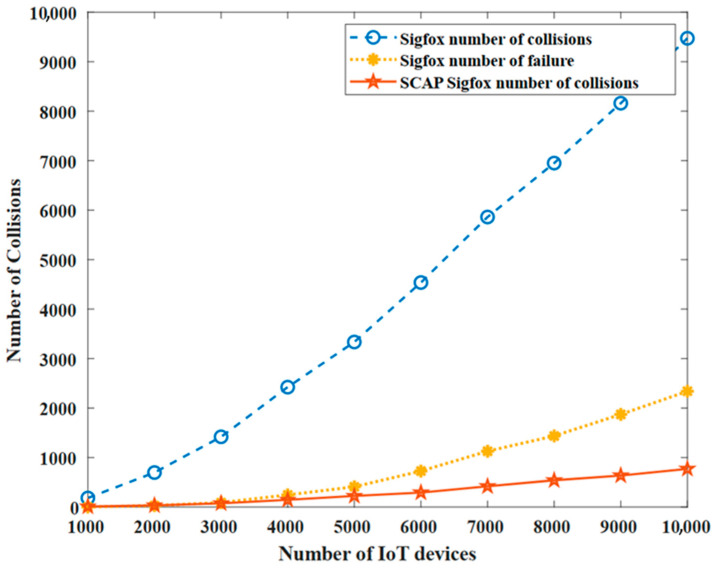
The number of collision and failure.

Figure 7 shows a comparison between SigFox and SCAP in terms of the number of collisions endured during the simulation time in a range from 1000 to 10,000 IoT devices. Additionally, it shows the number of failed transmissions in SigFox, which counts the number of messages where the three copies failed to be successfully received by the base station. First of all, as expected, the number of collisions increased with the number of nodes, as more nodes were competing to transmit data messages. 

Moreover, we can observe that the number of collisions in the original SigFox was much higher compared with the number of collisions in the SCAP SigFox since the original SigFox uses the three-times redundancy procedure while SCAP transmits a unique copy of the message in a unique orthogonal channel. 

**Figure 8:**
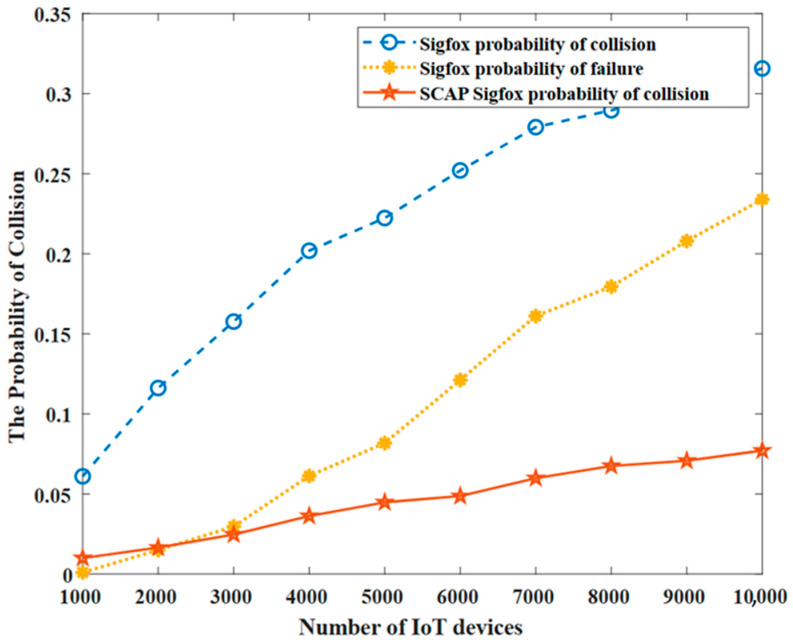
The probability of collision and failure.

In fact, the original SigFox uses the redundancy mechanism to ensure reliability, and hence a collision experienced by one or two copies of the message will not prevent the message from being successfully received by the base station as long as at least one copy is delivered. Therefore, failure is defined for the original SigFox only when the three copies of the same message experience collisions and hence no copy is successfully received by the base station. However, we calculate the collisions for the original SigFox by considering all the collisions for all the message copies. For instance, let us assume that a node sends three copies of the same message to the base station. If only two of them experience collisions, then the number of collisions is two and the number of failures is zero. On the other hand, in the SCAP solution, if a collision occurs then it is considered as a failure since no redundancy mechanism is employed as only one packet is transmitted using a pre-determined slot and channel identifier. Hence, in the SCAP method, collisions are failures. Figure 8 shows the difference between a collision and a failure in the original SigFox. Furthermore, it shows a comparison between the number of collisions and failures in the original SigFox and the SCAP method. 

Most importantly, SCAP achieved a lower number of collisions than the number of failed messages in SigFox. This proves that by combining the time division multiple access (TDMA) technique along with the slot- and channel-allocation technique we not only reduced the number of collisions but also increased the reliability of SCAP SigFox and, thus, the network scalability was improved as an extremely large number of nodes could communicate successfully. 

In our simulation, we considered other performance metrics to obtain a more accurate evaluation of SCAP SigFox such as the probability of collisions, the network throughput, the packet delivery ratio, and the energy per bit.

### 6.1. The Probability of Collision: P(C)

The probability of collision is calculated as the ratio of the total number of collisions or failed transmissions to the total number of transmitted packets by all the IoT devices. The probability of collision, P(C), is expressed as follows:

(7) $$P(C)=\frac{C}{n_{Trans}}$$

where C is the total number of collisions and $n_{Trans}$ is the total number of transmissions.

Figure 8 shows the probability of a collision in the original SigFox alongside the probability of failure as well as the collision probability of SCAP. We noticed a high rate of failure in the original SigFox due to the Aloha-based medium access protocol and the use of partially overlapping channels. Moreover, the probability of collision in the original SigFox was higher than the probability of failure, as failure was considered only when all three copies of the same message experienced collisions and hence there was no successful delivery of any copy of the message. Most importantly, it is worth pointing out that both the collision and failure probabilities in the original SigFox were much higher than the probability of collision in SCAP SigFox, where the probability of collision in SCAP did not exceed 0.1 when 10,000 IoT devices were transmitting. Due to this reduced range of the probability of collision in SCAP SigFox, we can anticipate that the throughput and PDR of SCAP will be improved compared to the original SigFox. 

Indeed, thanks to the use of only the 360 orthogonal channels in addition to our channel- and slot-assignment procedures, the probability of collision in SCAP was highly reduced. Recall that to carefully assign the orthogonal channels, SCAP SigFox divides the network field into tiny sectors with an angle of 1°. Thus, a small number of nodes share the same sector and hence the same channel. 

Moreover, to avoid collisions among the nodes on the same channel, a time separation is achieved using TDMA access, where slots are autonomously assigned to nodes. Consequently, the probability of collision is further reduced, which enhances the network scalability as a large number of nodes can successfully communicate under a small collision probability. 

Please note that, although improbable, collisions still may happen with SCAP SigFox, as two nodes may share the same slot if they are within the same distance from the gateway. 

### 6.2. The Throughput (TP)

The throughput is calculated as the amount of information (no redundant data messages) transmitted successfully during the simulation time. The throughput, TP, is expressed as follows:

(8) $$TP=\frac{S}{T_{sim}}$$

where S is the total successful non-redundant transmissions, and $T_{sim}$ is the simulation time.

**Figure 9:**
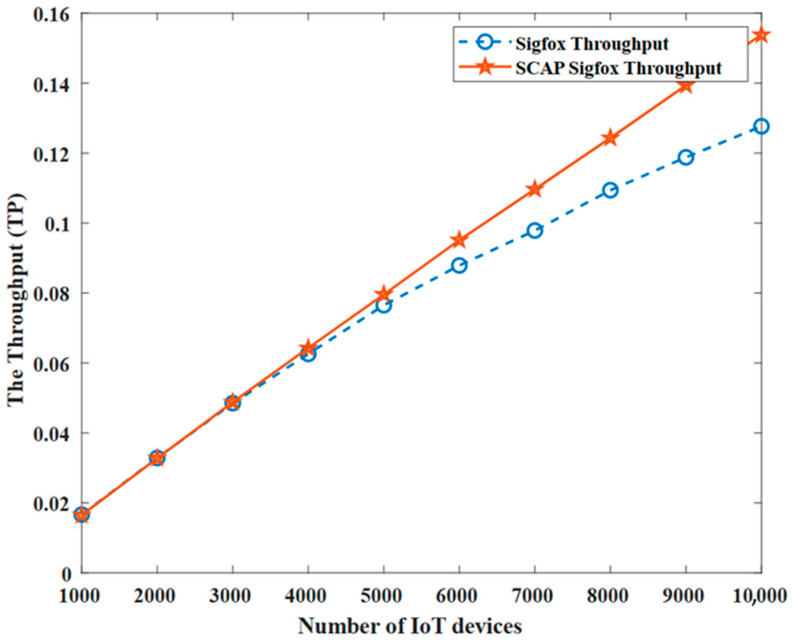
The throughput (TP).

Figure 9 shows a comparison between the original SigFox and SCAP SigFox in terms of the throughput. In order to achieve a fair comparison, the throughput for the original SigFox was calculated by considering only the non-redundant transmissions.

As shown, the throughput of SCAP SigFox was greater than that of the original SigFox, which demonstrates that SCAP SigFox was more efficient since the throughput is an indicator of the effectiveness of a network. This was achieved in SCAP SigFox simply by avoiding overlapping the channels and implementing TDMA along with slot- and channel-allocation techniques to prevent collisions.

### 6.3. The Packet Delivery Ratio (PDR)

The packet delivery ratio (PDR) is computed as the percentage of the successfully transmitted packets compared to the total number of transmitted packets by all the IoT devices. The packet delivery ratio PDR can be derived as follows:

(9) $$PDR=\frac{S}{n_{Trans}}$$

where S is the total number of successful transmissions, and $n_{Trans}$ is the total number of transmissions in the network during the simulation time.

**Figure 10:**
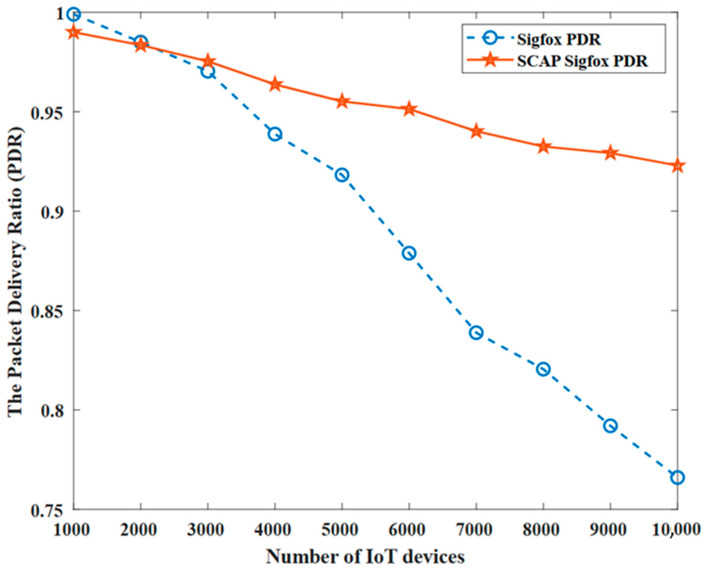
The packet delivery ratio (PDR).

Figure 10 shows a comparison between the original SigFox and SCAP SigFox in terms of the packet delivery ratio (PDR). The PDR for the original SigFox was calculated using only the non-redundant transmissions to ensure a fair comparison.

First, we point out that, as expected, the PDR decreased with the increase in the number of devices due to the probability of collision increasing. Most importantly, note that the PDR of SCAP SigFox ranged from 1–0.9, while that of the original SigFox ranged from 1–0.75, which indicates that the reliability of SCAP SigFox was higher thanks to the implementation of slot- and channel-allocation techniques. 

The fact that using TDMA-based medium access indeed increased the waiting time of a message to access the channel; however, once the message in SCAP SigFox was transmitted it had a 91% chance of successful reception and, hence, the probability of time- and energy-consuming retransmission was reduced. Meanwhile, the original SigFox uses Aloha-based access media, which indicates that the waiting time was indeed zero, but the messages had only a 76% chance of successful reception due to the random transmission that resulted in a high collision rate and, hence, retransmissions were needed, which negatively affected the network’s performance, especially in terms of PDR and throughput.

### 6.4. The Energy Consumption 

To compare the energy consumption in our simulation, the energy/bit value was calculated, which can be described as the ratio of the total energy consumed in the network to the total number of successfully received data bits. The energy per bit, $E_{bit}$, is expressed as follows:

(10) $$E_{bit}=\frac{\sum \left(consumed\;energy\right)}{S*L}$$

where ∑(consumed energy) is the total energy consumed by all the nodes during the simulation time, S is the total number of successful transmissions, and L is the packet length in bits.

**Figure 11:**
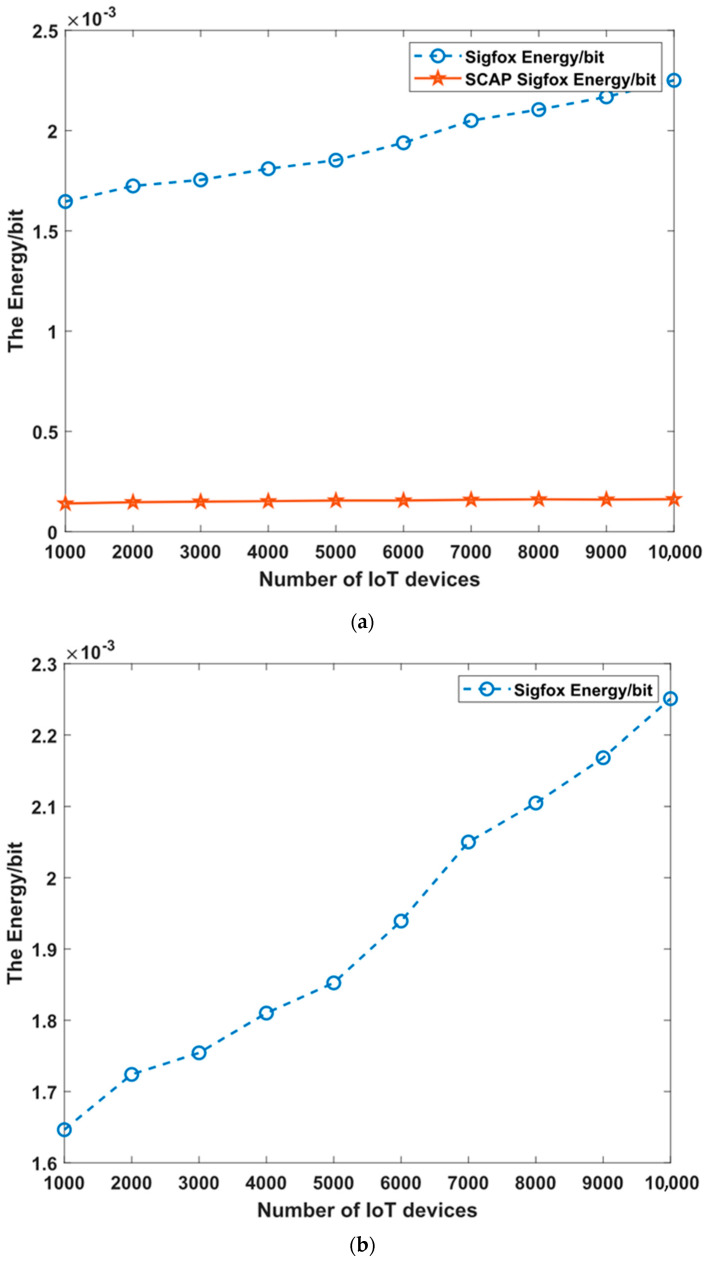

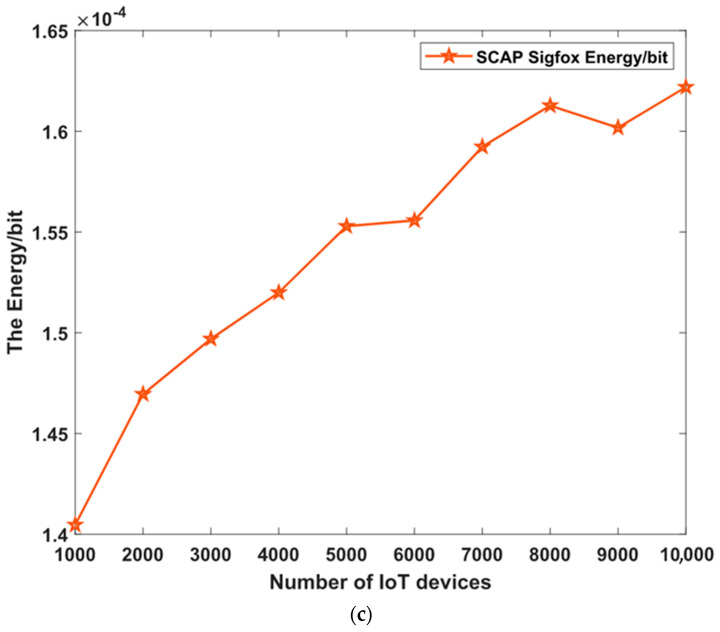
(**a**) The energy/bit value of original SigFox vs. SCAP SigFox; (**b**) the energy/bit value of original SigFox; and (**c**) the energy/bit value of SCAP SigFox.

Figure 11a shows the energy/bit value of the original SigFox vs. the SCAP SigFox. Moreover, two more figures, namely Figure 11b, presenting the energy/bit value of the original SigFox, and Figure 11c, presenting the energy/bit value of SCAP SigFox, are shown for clarification and comparison.

As observed in Figure 11, the energy per bit value for the original SigFox increased to reach approximately 1.75 J/bit, while for SCAP SigFox, the energy increased but at a much lower rate, reaching 0.129 J/bit. Evidently, SCAP did indeed reduce the energy consumption due to not only the reduced number of collisions and, hence, the reduced number of retransmissions but also mainly thanks to the transmission of a single copy for each data message as opposed to the original SigFox, where three redundant messages are transmitted for every generated data message.

## 7. Conclusions

In IoT networks, simple mechanisms are used to increase the probability of successful message delivery such as message redundancy. These mechanisms are not efficient enough as they can increase the number of collisions in the network and, thus, long-range wireless IoT networks cannot be reliably used [24]. To improve the network reliability and scalability, we proposed the slot- and channel-allocation protocol (SCAP), which is an autonomous collision-prevention protocol. 

SCAP SigFox uses two autonomous allocation mechanisms: time slot and channel assignment. The main goal is to assign a specific time slot and channel identifier to each IoT device so as to guarantee conflict-free channel access. These two assignment procedures are fully autonomously calculated by the IoT device without requiring any communication with the base station to avoid creating a bottleneck at the base station and to ensure efficiency in duty-cycled networks. Once SCAP SigFox is implemented, each IoT device predetermines the slot identifier and the channel identifier based on its geographical location and its distance from the base station. The message is then only transmitted once (rather than three times) on the assigned time slot and the selected channel. 

Furthermore, MATLAB simulations were conducted to evaluate SCAP SigFox compared to the original SigFox in terms of the number of collisions, the number of failures, the probability of collision P(C), the probability of failure P(F), the throughput (TP), the packet delivery ratio (PDR), and the energy consumption (E). The simulation results proved that SCAP SigFox improved the collision rate by 82% and the throughput by 10.9%. Furthermore, SCAP SigFox was more reliable than the original SigFox, as the PDR ranged between 91–99%, while the PDR of the original SigFox ranged between 76–99%. Finally, SCAP SigFox was much more energy efficient, as it reduced the energy consumption by 92% due to the elimination of redundancy and retransmissions caused by collisions.

Finally, the slot- and channel-allocation protocol (SCAP) resulted in a significant reduction in the number of collisions compared with the original SigFox, thus making SCAP a more scalable solution, as demonstrated experimentally. 

In future work, it would be interesting to conduct a simulation-based comparative study with other protocols that enhance SigFox, especially by using TDMA as the medium access mode [25,26]. Additionally, we will work on improving the maximum frame size (m) calculation, as it is driven, currently, in our SCAP solution from the network field size and the network density. By finding the optimal calculation of m, we could find the exact number of needed slots in a frame and, hence, achieve a further reduction in the number of collisions. Furthermore, the current slot assignment mechanism is a distance-based solution relying on the average distance (d) between the IoT devices in a circular field to assign slots. As an improvement, we want to investigate and find the optimal distance (d) according to which the slot assignment mechanism is completely conflict-free.

## Figures and Tables

**Table 1:** Parameter settings.

Simulation Parameter	Fixed Value
Number of IoT devices	1000 to 10,000 IoT devices
Bandwidth	192 kHz
Channel size	100 Hz
SigFox number of channels	1920 channels
SigFox orthogonal channels	360 channels
Transmission time	2 s
SigFox number of packets	3 packets
SCAP number of packets	1 packet
Message payload	96 bits
Transmission power	25 mW

## Data Availability

The data presented in this study are available in the article.
